# The Affective Domain—A Program to Foster Social-Emotional Orientation in Novice Physical Education Teachers

**DOI:** 10.3390/ijerph18147434

**Published:** 2021-07-12

**Authors:** Sima Zach, Hily Rosenblum

**Affiliations:** School of Graduate Studies, The Academic College at Wingate, Netanya 4290200, Israel; hily@wincol.ac.il

**Keywords:** social-emotional orientation, physical education, professional development program, personal development

## Abstract

The present study aimed to assess the influence of an emotional-based program for novice physical education teachers on their perception of the affective domain in teaching, and the influence of the program on their social-emotional orientation. Thirty-two physical educators in their induction year participated. Instrumentations included reflective assignments: individual tasks, a group artwork task, short videos containing student–teacher scenarios, and summary reflections. The study covered tasks that contained a variety of emotional expressions—verbalizing, acting, and art creation. Content analysis was conducted for each of the assignments. The results indicate that the participants felt that they gradually developed an awareness of the role of emotions in their practice. In addition to personal gain, they felt that their empathy for others—especially their students—was enhanced. These results highlight the important influence that an emotional-based program has on physical educators’ social-emotional orientation.

## 1. Introduction

The affective domain is part of the human inner world, as well as outer world relationships and communication with others, with implications for the individual’s well-being and quality of social relationships [1,2]. Enhancing the ability to perceive the self and others’ emotions requires social-emotional intelligence from every individual, and all the more so from teachers who are responsible for their class as a microcosmos [3].

The emotional environment of a class is of great importance to the development of both students and teachers [4,5,6]. Their interpersonal relationships have a mutual influence on the overall class climate, learning and teaching effectiveness, motivation, and well-being [7]. However, while students’ emotional worlds have been extensively documented, the affective domain of teachers is relatively undertheorized [3] and underexplored [8].

Along the lines of the Organization for Economic Co-operation and Development (OECD) document concerning education in 2030 [9], we believe it is of great importance to equip teachers with the knowledge, values, attitudes, and skills needed to educate current students who will be young adults in an uncertain future. According to this document, the building blocks that create the framework of learning contain four domains: cognitive and meta-cognitive skills, physical skills, attitudes and values, and social-emotional skills. Currently, we are facing a gap between what we teach and what is needed, and we identify with the OECD target that declares social-emotional learning as one of the building blocks that should be fostered. In the current study, we chose to examine ways to foster social-emotional skills among teachers, due to their relatedness to their well-being, success, and connectedness, and thus, to the increase in the likelihood that they will be able to instill these skills in their students. According to the OECD definition, the term “social-emotional skills” refers to the ability to regulate one’s thoughts, emotions, and behavior [10]. These skills are dependent on situational factors and are responsive to change and development through learning experiences. They have been shown to influence many important life outcomes, and also to influence the development and use of cognitive skills.

It should be noted that social-emotional aspects, such as sportsmanship, teamwork skills, trust, resilience, and other non-physical attributes important to sports and physical education, are inherent to team and group activities, and are fundamental requirements for successful performance. Nevertheless, PE teachers traditionally place greater emphasis on enhancing and developing aspects of the physique, and in order to do so, they mostly use instruction and practice teaching styles [11].

To reach this target, teachers should develop the ability to build an atmosphere that will enable them to acquire such skills. Traditionally, in physical education teacher education programs, the emphasis has been on the physical domain. The social and emotional aspects were negligible or narrowly developed and almost absent from the training program, as can be seen in the main goals of several national physical education curricula in the previous one or two decades [12]. Hence, when beginner teachers encounter difficulties in school that are social-emotional-related, they feel helpless and frustrated [13]. Thus, we found it of paramount importance to implement a missing chapter in teacher education programs—that is, to develop a series of sessions on the development of social-emotional learning in beginner teachers. Teachers in practicum (TPs) were chosen as participants because their overwhelming social-emotional interactions with learners, colleagues, parents, and management—"the real world”—do not appear in any textbook. They can bring these cases to the course, which can serve as the best laboratory for studying according to their needs.

In the current study, the social-emotional intelligence model of Bar-On [14] was chosen as the conceptual framework for attempting to bridge the gap of knowledge regarding the affective domain of teachers. Specifically, the affective domain of physical education teachers, whose teaching setting—although physically oriented, conveys countless situations that demand social-emotional intelligence. We chose this model because it was based on a questionnaire that is suitable for individuals aged 17 years and above, measured in a school setting or in a workplace of young adult populations, and deals with factors regarding its impact on physical health, psychological health, self-actualization, and subjective well-being [15,16,17,18].

The social-emotional intelligence model is comprised of interrelated emotional and social competencies and skills that determine the effectiveness of how people understand and express themselves, understand and relate to others, and cope with daily demands. Bar-On [14] developed the EQ-I Scales to measure the social-emotional intelligence construct, which includes five scales: intrapersonal, interpersonal, stress management, adaptability, and general mood. These scales assess five core social-emotional competencies: (1) self-awareness and self-expression—the ability to recognize one’s own feelings, interests, and strengths, in addition to maintaining an accurate level of self-efficacy; (2) social awareness and interpersonal relationships—taking others’ perspectives into account and empathizing with others, developing and maintaining healthy relationships with others, including the ability to resist negative social pressures, resolving interpersonal conflicts, and seeking help when needed; (3) emotional management and regulation—the ability to handle daily stresses and to control one’s own emotions under difficult situations, including the ability to monitor and reflect on personal and academic goal-setting; (4) change management—the ability to adapt and adjust one’s feelings and thought processes to new situations, and to identify problems and develop appropriate solutions to those problems; and (5) self-motivation—to be positive and optimistic, and to feel content with oneself, others, and life in general. This model was validated by others who affirmed that social-emotional intelligence competencies and skills can be trained and fostered (e.g., [1,14,19,20,21]).

In a meta-analysis study that examined the implementation of social-emotional learning programs in schools, Durlak et al. [22] reported better social-emotional results among students who participated in such programs compared to students who did not. Specifically, the former demonstrated increased academic achievement and social-emotional skills, improved attitudes toward the self and others, positive social behaviors, and decreased conduct problems and emotional distress. These results were consistent across grade levels, locations of the schools, and school types.

Research concerning the role of the affective domain in teaching and in teacher education (e.g., [23,24]) revealed a significant gap between declarative knowledge and perceptions of the importance of educational themes—such as the affective domain—to the actual behavior or to behavior changes in this domain (e.g., [25]). When considering emotions as the mediators between knowledge and behavior [26], we found the affective domain to be a venue of research that can provide practical implications to teacher education programs and to teachers’ professional development programs.

Traditionally, in physical education, the curriculum aims, themes, and content have been pervasively oriented towards sports-dominated programs, in which competitive activities have a significant role and the educational goals are focused mainly on developing the students’ motor abilities [27,28]. The cognitive domain was always part of the education process, but only toward the 21st century did it take the lead in national physical education programs (e.g., [9,29,30]. The current study adopted a holistic approach and followed others researchers who suggested that several domains should be interrelated during the process of becoming physically and literacy-educated (e.g., [31,32]). In other words, teacher preparation and professional development training should include detailed programs that, in addition to promoting the motor and cognitive competencies and skills, emphasize the affective domain with its emotional and social aspects.

Using art therapy tools promotes mindfulness and the expression of feelings [33], which leads to holistic self-perception, acceptance of others, and empathy [34,35]. Research has shown that using art therapy in schools assists in dealing with emotional difficulties (e.g., [36,37]), and leads to a decrease in aggressive behavior [38] and characteristics of attention/concentration problems [39]. In their meta-analytical review on studies that examined the implementation of art therapy in the Israeli education system, Snir and colleagues [40] recommended that teacher education programs provide teacher candidates with courses that will equip them with the understanding and the ability to use art therapy tools. Along these lines, we chose to apply art therapy in physical education teacher education—an approach that is recommended due to its orientation towards developing a humanistic and interactive teaching approach [40,41]. Hence, the current study aimed to examine the influence of a multi-stage program on the enhancement of the affective domain in physical education teacher education. More specifically, its goal was to examine the impact of the program on novice physical education teachers’ social-emotional orientation—including their competencies and skills.

## 2. Methods

### 2.1. Participants

The participants were 32 first-year novice teachers who are teachers in practicum (TP), including 9 males and 23 females. Their age range was 24–29 years; 13 worked in elementary school, 8 in junior high school, and 11 in high school. In Israel, upon completion of 80% of the curriculum of their teacher education program, student teachers are permitted to become teachers. The participants were members of the school staff and received their salaries from the Ministry of Education. They were recruited from a course entitled “Case Studies in Teaching”, designed particularly for TPs, and mandatory for all student teachers in order to receive their Bachelor of Education (B.Ed.) degree in physical education and a teaching license. The course has a flexible syllabus and is adjusted according to the participants’ needs. All of the meetings are conducted as workshops in one semester. After all the participants gave their informed consent, the investigators began examining the course. It should be noted that participation in the study was on a voluntary basis. The participants could choose the group they wanted to study among three possibilities that were given in the same time slot and under the same course title. The study group was one among these three. At the first meeting, they were invited to participate in the study and sign consent forms or, if they preferred, to change to another group.

### 2.2. Instrumentations

Reflective diary. A total of 19 reflective tasks were collected and recorded in a diary kept by each of the participants. The diaries were designed to document the participants’ personal and professional voyages during the program.

Art creations. Art creations are an example of an in-depth application of a phenomenological research approach used in art therapy [42]. This approach leaves the observer/researcher open to describing the studied occurrences and promoting discussion for wider understanding. Art creations produced by the participants throughout the sessions were based on intuition and insight, and their analysis provided an understanding of their perceptions, as suggested by others (e.g., [43]). Participants’ verbal descriptions of their experiences through photographs of expressive art products involved in their personal and professional development served as a significant source of data for subsequent systematic analysis. Art activities done throughout the program, along with verbal descriptions of the experience, contributed to the understanding of the meaning that the participants give to their experience. Art activities hold potential for releasing emotional barriers through metaphors and non-verbal modalities for expression—plastic art, movement, and drama. In this visual creation, metaphors are used for understanding one’s perceptions [44]. The researchers supported the use of metaphors to enhance the educators’ ability to express their feelings freely [45].

Role-playing activities. Visual arts, including painting, sculpturing, film making, and theatre, offer a multi-dimensional means for the expression of thoughts and feelings [46,47,48]. Therefore, we observed and analyzed role-playing activities that were filmed on video, as well as photographed throughout the sessions of the course, aiming to capture the actors’ expressions, the story that was being told, their feelings, and the verbal explanations that followed. In addition, the participants were asked to choose photographs that metaphorically best expressed their interpretation of what they had learned throughout the course, and to reflect upon them. Expression through role-play, art, music, and movement, can elicit data concerning people’s ways of communicating without words, and serves as a rich research tool both in therapy and education.

An exhibition and reflective summary. All the participants were required to choose how and what to express in the assignments that they submitted along the timeline of the course, and to present some of the assignments in a final project—the course exhibition. This display triggered intentional observations that enhanced the awareness of the messages embedded in the artistic expression, which has been termed the “phenomenal field” [49]. In addition, the participants were invited to walk around, observe, and then submit an assignment, which was a reflective summary of the course. This integrative reflective task allowed us to identify the stages of development, analyze each stage separately, and understand the impact and contribution of each stage to the whole process.

Table 1 summarizes the workshop stages with the instruments and type of data collected in relation to the social-emotional intelligence aspects that were developed.

### 2.3. Procedure

The research began after obtaining approval from the college institutional review board (IRB), and after each participant signed a consent form. The course was defined as the intervention part of the study and aimed to increase the teachers’ self-expression skills, the ability to identify their own feelings as well as those of others, and to effectively regulate them. The course program is based on holistic/humanistic theories (e.g., [50,51]), and on the Expressive Art Therapy field, aiming to generate attention to the personal dimension and the expression of emotions via art modalities [49,52,53,54,55].

The course was designed as a workshop and included the following assignments: (a) documenting a challenging teaching event focusing on body sensation, emotions, and thoughts; (b) documenting a challenging dialogue between a teacher and student, focusing on group activities that included writing the scenarios of these dialogues, acting according to the script, and filming the scenario; (c) reflecting on the challenging dialogue in the video-filmed scenarios; (d) preparing group artwork that expresses the challenging scenario that was filmed; (e) reflecting on the challenging dialogue based on art activities focusing on metaphor expressions; (f) recording integrative reflective insights of the course work.

During the meetings, several activities took place, as follows:

(1) Documentation of a challenging teaching event focusing on body sensation. This part had several activities: (a) The participants’ home assignment was to document a challenging event that they encounter in their teaching; (b) in class, they had to report emotions, body sensations, and thoughts; (c) in the same lesson, they were divided into groups of four people—the teller described his/her event to another participant in the group; the listener, who was instructed to listen with no comments, while the other two people—the photographers, filmed the listener and the teller, each one from a different angle; (d) the group observed the video clips and each member added details that he/she thought would be significant for their first documentation; (e) the participants were asked to rank the following three aspects: emotions, body sensations, and thoughts, according to their dominant appearance.
(2) Body sensations, emotions, and thoughts. In the next lesson, the TPs were divided into three groups based on the dominant aspect that they recognized in their own documentation—emotions, body sensations, or thoughts. Their assignment was to express verbally, as well as non-verbally, the dominant aspect of their group, using photographic art material. By integrating these three aspects, that is, emotions, body sensations, and thoughts, we aimed to arrive at a holistic perception of a challenging event.
(3) Documentation of a challenging dialogue of a teacher and student, focusing on group activities by verbal and non-verbal means. The TPs chose two photographs of trees, metaphorically representing a teacher and a student. Using these two pictures they created a poster symbolizing a challenging dialogue between them.
(4) Reflective observation. (a) The TPs wrote down the challenging dialogue that emerged from the previous activity; (b) in the next activity, which was filmed, this dialogue was used as a scenario for a simulation between a teacher and student.
(5) An analysis of the challenging dialogue in teaching, focusing on practical insights. The TPs watched the video clip twice. The first time, they observed the simulation and documented the body sensations, emotions, and thoughts, and the second time, they observed the video clip on mute mode and wrote a dialogue for this challenging event; these two activities were then discussed in class for developing practical implications.
(6) Integrative reflection. The novice teachers were required to fill out and submit a short feedback form relating to their perceptions about the course’s contribution to their professional development. Some of the questions were “What were the meaningful issues in the course?”, “How do you perceive the course activities’ impact on you?”, and “Can you provide any feedback or suggestions?”.

### 2.4. Data Analysis

We applied several techniques, following Inayatullah’s [56] Causal Layered data analysis. The first one was word counting. Ostensibly, this technique is more related to the quantitative research approach. However, it has been used by other qualitative researchers to display the presence of certain words, which indicates a repertoire or a lexicon that reflects patterns and insights of thinking, as well as emotional constructs that can be extricated from counting [57].

Second, we extracted the metaphors used from their verbal and visual artwork. Lakoff [58] and Lakoff and Johnson [59] developed the theory of the conceptual metaphor. According to this theory, metaphors are cognitive constructs that enable the conceptualization of complex domains, using simple domains known from everyday experiences. Therefore the usage of metaphors in discourse is frequent. Other, discourse-oriented researchers emphasized that metaphorical patterns derived from a discourse reflect the integration of thinking, emotion, society, and cultural aspects [60,61]. Metaphorical language is common in the spoken, written, and online discourse, and it indicates people’s thoughts, feelings, and culture. We followed the discourse dynamics method of metaphor analysis suggested by Cameron and her colleagues [60] that continuously moves across three levels and timescales: the micro-level of a particular metaphor, the meso-levels of episodes of talk or topic threads, and the macro-level of the conversation as a whole, with the broader socio-cultural level. After the data was prepared, the metaphors were identified and then coded for patterns that yielded information about the participants’ ideas, attitudes, and values [60].

Third, we compared the patterns of words and metaphor usage between assignments—from the beginning of the course to those handed out at its end. Fourth, content analysis was conducted, following best practices recommended by others (e.g., [62]), on the verbal components of the visual data of the simulation videos and on the verbal description of the art created by the creator.

### 2.5. Trustworthiness

We followed Lincoln and Guba’s [63] claim that trustworthiness involves the establishment of four elements: credibility—confidence in the “truth” of the finding; transferability—showing that the findings can be applied in other contexts; dependability—showing that the findings could be repeated; and confirmability, showing that the findings of a study are shaped by the respondents and not the researcher. We achieved credibility through several actions: prolonged observation, researcher and several data sources’ triangulation, peer debriefing, and member checking [64]. Each of the two authors performed the analysis independently. Then, a discussion was held about the themes until full agreement was reached. Member checking was conducted at the end of the course. A presentation with the analysis was held, and all the participants were invited to relate to the themes. Most of them added examples to support the themes. No additional theme emerged at that stage.

Transferability was achieved using “thick description” [63]. By describing our activities in sufficient detail, one can evaluate the extent to which the conclusions drawn in the current study are transferable to other times, settings, situations, and people. All the details needed in order to repeat the study can be obtained from the vivid picture of the research procedure.

Our activities, to enable us to achieve confirmability, included an audit trail, three types of triangulation [65,66]—method triangulation, source triangulation, and analyst triangulation—and reflexivity. We developed reflexivity by designing research that included multiple investigators. This fostered dialogue and provided a context in which our perceptions and assumptions could be revealed. In addition, since we were focusing specifically on language that is related to thoughts and emotions, the researchers were given the responsibility of reporting on our intervention, which they did. The metaphors that were chosen for this article were solid and rich with meaning and demonstrated the verbalization of either the experience of emotions or the expression of emotions.

## 3. Results

While analyzing the content, we noticed that the TP gradually developed the participants’ ability to recognize emotions and to express and deal with them. We outlined six stages that best characterize this developmental process. Within the stages, we describe the characteristics of the TP expressions and outline examples of episodes that were described by them.

(1) Recognition and awareness concerning the role of emotion. At the onset of the course, the documentation of challenging events and the analysis and counting of words describing pleasant and unpleasant inner- and inter-interactions were performed. In this stage, we encountered pleasant and unpleasant emotions that the participant referred to in their inner world, as follows:

Pleasant emotions (13)—happiness, pleasantness, encouragement, thankfulness, helpfulness, love, hope, respect, praise, relief, liking, and feeling secure.

Unpleasant emotions (15)—nightmares, difficulties, disgust, resentment, fear, anger, discrimination, sadness, ignorance, boredom, helplessness, despair, embarrassment, anxiety, and panic. For example,

*“I feel so confused. I am trying to explain, but all of the students are talking together, there is so much noise that I cannot hear anyone, and not give any feedback… I am in a panic.”*

Unpleasant emotions that the participants felt towards others (4)—rejection, denial, urge/need to prove, and contempt.

Emotions referring to their inner experiences (2)—excitement and acknowledging their feelings. For example,

*“Because I think all the time about what I have done wrong, and how to change, I feel restless and impatient”.*

Emotions concerning experiences with others (7)—caring, excitement, relatedness, admiration, belonging, appreciation, and closeness.

(2) Conceptualization of emotional experiences. Following their vocabulary usage, we noticed that the ability to conceptualize emotional experience had developed:

Pleasant experiences (5)—happiness, positive experience, success, laughter, thinking healthy thoughts.

Unpleasant experiences (12)—sorrow, nervousness, anger, loss, unpleasantness, fear, fright, helplessness, anxiety, difficulty, frustration, unsuccessfulness.

Emotions concerning others (3)—contempt, connectedness, admiration.

Emotions concerning the self (4)—body awareness, self-awareness, low self-esteem, negative self-esteem. For example,

*“I know that I am disappointed and angry about my students, but with a deep breath and patience things will get better.”*

**Figure 1:**
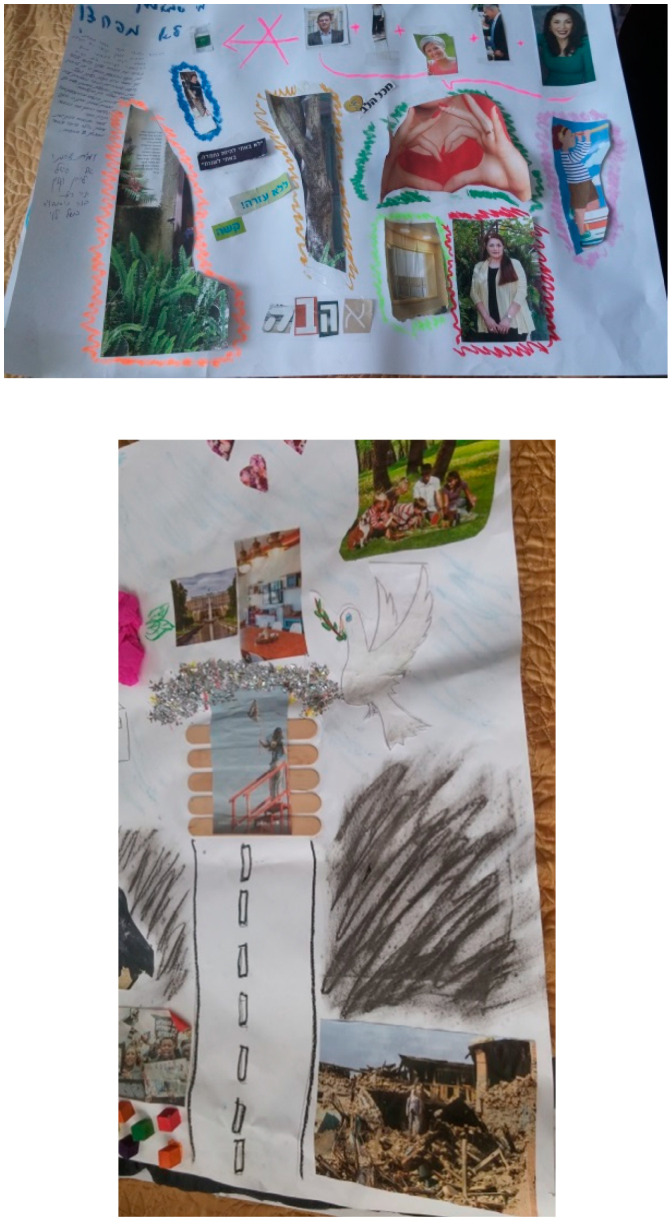
Examples of group art.

It appeared that the group work activities allowed openness, so the participants gradually felt comfortable enough to express the more authentic unpleasant words that expressed their more unpleasant feelings. Figure 1 presents the group art that required dialog, collaboration, and openness.

**Figure 2:**
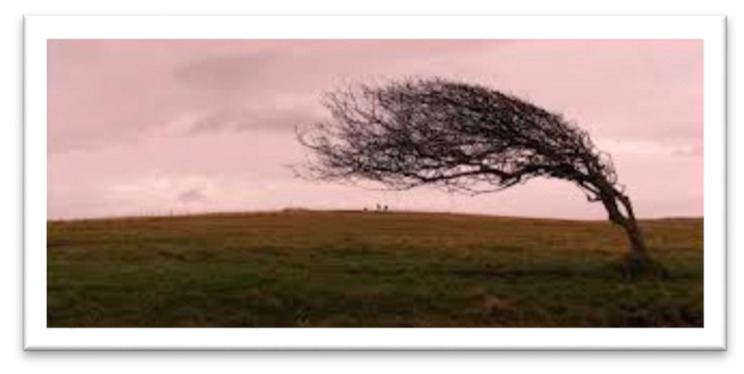

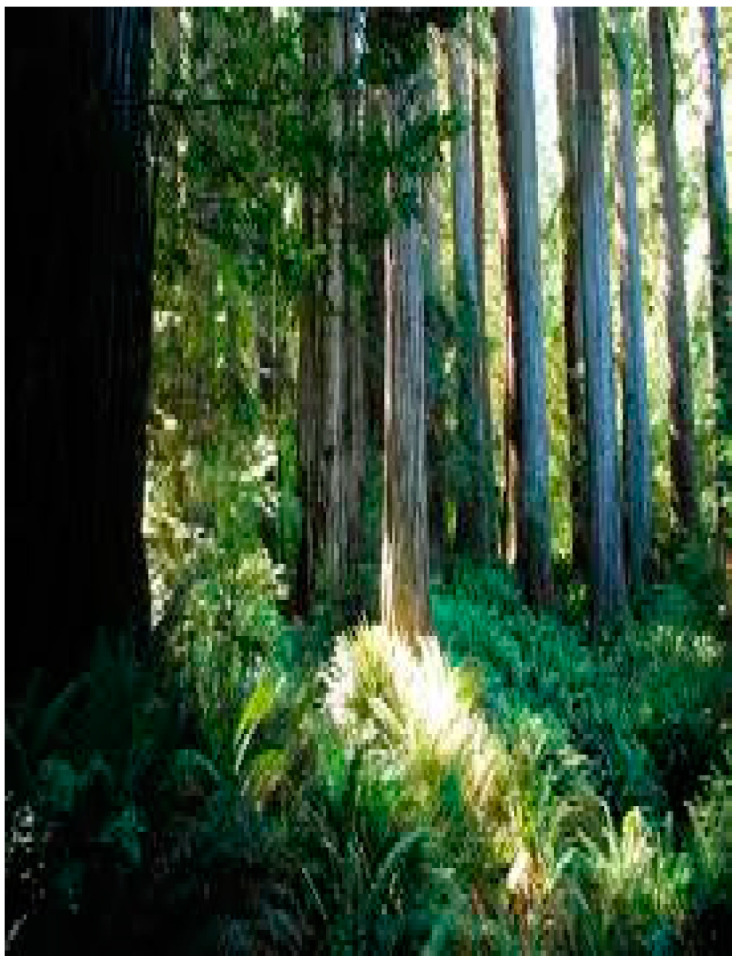
Examples of trees.

(3) *Rich and metaphoric emotional vocabulary.* When the metaphor assignment was presented, it was noted that the participants expressed their emotions freely and richly. The metaphor assignment required reflection on a challenging event using a wide and holistic approach. The TP had to choose a picture of a tree that best described their feelings regarding a particular event. Figure 2 depicts examples of trees that were chosen.

It should be noted that not all the novice teachers had reached this level of ability to express emotions at this stage. Some were still puzzled:

*“What do they want from us?”*

This is an example of behavior reflecting a novice teacher who still found him/herself located at the personal stage, who is both a student and a teacher, and still is not able to perceive the role of the teacher concerning teaching–learning events.

In this stage, the TPs used 34 pleasant words and also 23 pleasant expressions, such as,

*“What one sees from one point cannot be seen from another place”, “you and I are gonna change the world”, “give me and I will give you”, “we are together all the way”, “educate the child according to his/her own path”, tomorrow is a new day.”*

Unpleasant words and expressions (7), interpersonal experience words and metaphoric expressions (43), and interpersonal expressions (18) were also used.

(4) Developing a new approach to teaching. At this stage, the program’s focus on creative activity bore fruit. Writing a challenging dialogue between the teacher and the students enables the release of an expression of the “automatic”/naturalistic behavior of the teacher. This activity exposed the emotional reaction without emotional management, and then, when reflection was required, the TP succeeded in identifying unsuitable behavior that was neither effective nor contributed to a pleasant atmosphere. Hence, it was evident that the TP developed their own approach to teaching, based on dialogue and empathy toward their students. As one of the TPs said to her student,

*“At the end of the lesson, we both will stay together and talk.”*

In other words, the teacher’s behavior is based on a holistic vision of the situation, enabling a variety of possibilities as opposed to a fixed and immediate solution.

The role of the teachers as it appears in the written reflections illustrates authoritative but defensive teachers, which may even evoke a survival mechanism. They appear helpless to their students since they are occupied with searching for assistance from external factors, such as the school principals, or from the norms of school punishment, such as removal from the classroom. Such examples reflect the difficulties that the teachers encounter with, and their impact on, the students’ behavior.

Reflecting on the situation enabled the TPs to understand the event as a constructive part of the relationship between the teachers and their students. The TPs were exposed to criticism, which inhibits communication between teachers and students. They were exposed to the question “why” and the phrase “this is not the first time…”, which induced feelings of shame—a feeling known to lead to rejection, lack of cooperation, and/or even anger and nervousness in the dialogue.

In another example, a student threatened to throw a bottle at the teacher. The teacher tried to keep himself safe—

*“Come over here! Relax!”*

It seems that the teacher was responding to the threat of violence but did not attend to the inner feeling of the student the anger. Hence, he demanded that the student stand far away from him:

*“Stand on the side of the room”,*

and then wished to regain control of the situation, saying,

*“At the end of the lesson, we will discuss it again.”*

This reflection presents the attention of the teacher to the emotional experience of the child, with the understanding that the teacher’s behavior probably had an impact on the child. The teacher’s intonation, and his decision to keep the student in the class and discuss with him what happened later, shows his empathy toward the student.

The simulation technique served as an opportunity to release emotion, hence providing a wide base for reflection.

(5) Practical insights, simulation observation, and group case study analysis. At this stage, the participants watched the video clips twice—with sound and in a mute mode. This activity led to a group discussion in the class for developing practical insights. Watching the video a few times in the mute mode enabled them to focus attention on the nonverbal impact of body language. The participants dedicated time to in-depth reflection, and a variety of emotions were later expressed. At first, while watching their own video simulation, the participants were embarrassed. For example, the TPs said to the group,

*“Oh, I don’t want us to watch it... it is embarrassing … no don’t screen mine.”*

Later, they started to offer creative insights towards their peers’ simulation, for example,

*“You should look at his eyes...”, “look how you hold your hand…”, “how about trying a different approach…”, “look at the child…”, “how about talking to him in a different manner …”*

The discussion enabled the participants to delve into challenging moments that they have encountered in real time at school, with a broader perspective and in a safe, containing atmosphere.

(6) Integrative holistic development. In the last stage, the participants were asked to review and reflect on all five stages of the course. This procedure included an “exhibition tour” of the artworks produced throughout the sessions. In this stage, the participants were given the opportunity to encounter works that they had personally created as well as the group work. This procedure was followed by written feedback aimed at sharing their perceptions about the course’s contribution to their personal and professional development.

Content analysis of the questionnaire revealed the following results. The TPs ranked their satisfaction with the course as 5 on a five-point Likert scale. The mean score was 4.1 (S.D. = 0.63). Regarding the verbal feedback, they appreciated the course as contributing mainly to three aspects:

(1) Their understanding of the importance of dealing with the emotional domain in physical education. One TP, a 32-year-old male middle and high school teacher wrote,

*“When we were student teachers, we were mostly occupied with pedagogical content knowledge and with content knowledge; the learners’ feelings were seldom discussed. So, I have not invested time towards this aspect. Actually, I think this aspect is the basis for learning and communicating.”*

(2) Their ability to identify students’ feelings, as well as to express their own feelings with openness. One TP, a 26-year-old female high school teacher wrote,

*“They hated to run. Eventually, I understood that in order to motivate them, I should be smart rather than getting angry at them, and turn their hate into love.”*

(3) Their ability to include the emotional aspect while planning. One TP, a 27–year-old female elementary school teacher wrote,

*“I know that I want to see happy children with self-confidence, therefore I spend a lot of time with my mentor and on the Internet to find suitable activities. I understand this better now than ever before.”*

## 4. Discussion

The present study aimed at assessing the influence of an emotional-based program for physical education teachers on their perception of the affective domain in teaching, and the influence of the program on their ability to express their emotions concerning reported cases, so that emotions would be part of the discourse in their practice. It was expected that the emotion-based program would develop a holistic integrative teaching approach among novice teachers, and foster their social-emotional orientation. Novice teachers in the current study reported their perception that they had gone through a process of change. Unlike behavior that reflects criticism and a judgmental approach, which leads to emotional inhibition and defensive behavior, the current program encouraged participants to practice self-expression. Along the course, from their heightened recognition and awareness, the participants felt that they gradually increased their ability to be empathetic with their students, to share and express emotional experiences, to share their dilemmas and conflicts with peers, and to admit when they encountered difficulties or provide help when they identified others’ difficulties. These competencies and skills are characteristics of social-emotional intelligence, as described in Bar-On’s [14] model of social-emotional intelligence. Along the lines of the holistic/humanistic approach, which claims that the affective domain is an integral part of education [67], our results demonstrate that an intensive emotional-oriented program has the potential to change not only teachers’ perceptions, but can equip them with tools so that their behavior can be changed as well.

Throughout the course, focusing the attention on the participants’ emotional and cognitive needs reflected the educational learner support approach [68], which was the anchor of the emotional-based education program in the current study. Such a process enabled them to identify, express, and accept the feelings of their peers, as well as their students’ feelings. The “voice my voice” technique of teaching was practiced, so that the novice teachers could express themselves and develop non-judgmental, empathic listening skills [69,70,71]. This approach concurs with the argument of other researchers (e.g., [72,73]) that teaching and learning involve cognition, emotion, physical sensation, and intuition, and this should be taken into consideration.

Similar to others who found that social-emotional intelligence can be fostered (e.g., [1]), the social-emotional intelligence model of Bar-On [14] was also reinforced in the current study. This means that the five competencies and skills that reflect a social-emotional intelligent teacher were observed, and that self-awareness and self-expression were demonstrated throughout the course assignments. Social awareness and interpersonal relationships were demonstrated through the simulations and conversations that were held. Emotional management and regulation were described in the reflection assignment along with the course case studies talks. The participants described the process of change that they went through until they acquired the ability to adapt and adjust their feelings and thinking to new situations, and to identify problems and develop appropriate solutions for them. They summarized the program as one that instilled them with self-motivation to be positive and optimistic and to be content with themselves, their peers, their students, and with life in general.

Several limitations of the study should be mentioned. First, the program that was implemented lasted one semester, with no follow-up. Therefore, the long-term changes of behavior in the participants could not be determined. Second, it should be kept in mind that the entire process of the intervention program was conducted as a simulation. Moreover, the applied part of the participants’ behavior change, meaning their emotional attitudes toward their students, was determined only by their reports. No observations were conducted of these participants in their real world—in their classes, while they were teaching, and so forth. Therefore, conclusions should be drawn with caution. Nevertheless, the first step in changing behavior is developing self-awareness and an awareness of others [74,75]. Observational assignments and self-filming with peer analysis of the implementation of the social-emotional aspects are warranted. Third, teacher education programs, as well as professional development courses, are fundamental for fulfilling the requirements of the profession and for the advancement of the individual (respectively) within the education system. Nevertheless, a “washed out” effect can occur [76]. In other words, what has been learned may fade over time. Still, this process is not inevitable. There are several conditions at school that may help in its inhibition, such as in physical education—principal support, the ability to team teach with peers from school, the ability to perceive control over content knowledge and acquire new methods of teaching, and being proactive in soliciting assistance [76]. Lastly, the program was conducted by a specialist on the topic of emotion in education. In order to assimilate such a program into teacher education, or into professional development courses, teacher educators would have to study the program in its entirety.

Following the promising results of the current study, it is recommended that professional training, as well as teacher education, deliberately integrate thoroughness programs that promote social-emotional orientation and intelligence competencies among their prospective teachers.

## Figures and Tables

**Table 1:** Workshop stages, the instruments, and the type of data collected.

Workshop Stages and Teaching Issues	Instruments	Data Collected
Documentation—of a challenging teaching event focusing on body sensation, emotions, and thoughts	Reflective tasks 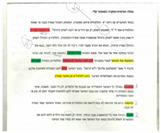	Descriptions of body sensation, emotions, and thoughts
Documentation—of a challenging dialogue of a teacher and student focusing on group activities	Art creations 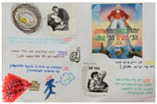	Verbal and visual means
Reflection—on the challenging dialogue based on art activities, focusing on metaphors	Written assignments relating to still photographic observations 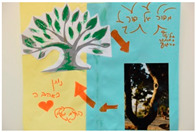	Metaphors
Reflective observation—of the challenging dialogue, focusing on video	Videos	Short films
Analysis—of the challenging dialogue in teaching, focused on practical insights	Individual reflective task	Practical implications drawn from the workshop analysis
Integrative reflection—student teachers’ feedback, practical awareness, insights development, and practical conclusions to implement in practice	Summary reflection	Integrating personal and group reflections

## Data Availability

Data are available upon request.
